# Superior prognostic value of soluble suppression of tumorigenicity 2 for the short-term mortality of maintenance hemodialysis patients compared with NT-proBNP: a prospective cohort study

**DOI:** 10.1080/0886022X.2020.1767648

**Published:** 2020-05-27

**Authors:** Zhiyu Wang, Zijin Chen, Haijin Yu, Xiaobo Ma, Chunli Zhang, Bin Qu, Wen Zhang, Xiaonong Chen

**Affiliations:** a Department of Nephrology, Ruijin Hospital, Shanghai Jiao Tong University School of Medicine, Shanghai, China; b Clinical Laboratory, Ruijin Hospital, Shanghai Jiao Tong University School of Medicine, Shanghai, China

**Keywords:** Dialysis, biomarkers, Mortality, N-terminal pro-brain natriuretic peptide, soluble suppression of tumorigenicity 2

## Abstract

**Background:**

Both soluble suppression of tumorigenicity 2 (sST2) and N-terminal pro-brain natriuretic peptide (NT-proBNP) are promising biomarkers associated with the adverse clinical outcomes of dialysis patients. Our research aims at exploring and comparing the roles of sST2 and NT-proBNP in predicting the short-term and long-term mortality of maintenance hemodialysis (MHD) patients.

**Methods:**

A prospective cohort study was performed. Patients undergoing hemodialysis in July 2014 were enrolled from the Blood Purification Center of Ruijin Hospital. MHD patients were followed up for 3 years. The primary outcome was all-cause mortality at the 1-year and 3-year follow-up, while the secondary outcome was cardiovascular mortality. Serum sST2 level was detected by quantified ELISA kits. Clinical data were analyzed by SPSS 23.0 version.

**Results:**

205 patients were recruited. The median sST2 level was 15.99 (11.60, 20.49) ng/ml. After 3 years of follow-up, both all-cause and cardiovascular mortality in 1 year and all-cause and cardiovascular mortality in 3 years increased significantly with serum sST2. For short-term mortality, no significant difference was observed in patients with increasing NT-proBNP levels. Cox regression analysis indicated that only sST2 was independent in predicting the risk of short-term outcomes. For long-term mortality, both sST2 and NT-proBNP were independent risk factors, while a higher hazard ratio was observed for NT-proBNP.

**Conclusions:**

Serum sST2 is a novel biomarker associated with adverse clinical outcomes in MHD patients. It was significant for both all-cause and cardiovascular mortality in MHD patients and may provide better prognostic value in short-term prognosis than the classic biomarker NT-proBNP.

## Background

End-stage renal disease (ESRD) has been a public health burden worldwide. The ESRD prevalence in Shanghai has increased to 898.2 per million population-years (pmp) in 2014 [1], while in United States it has increased to 2053.9 pmp in 2017 [2]. The mortality in ESRD patients is much higher than general population, especially in the first year starting dialysis treatment [3]. Fatal cardiovascular events were the leading causes of known cause-specific mortality, responsible for more than 40% of deaths in dialysis patients [2]. Hence, exploring new strategies to predict the risk of adverse clinical outcomes, especially cardiovascular disease mortality, may be beneficial to improve the outcomes of MHD patients.

N-terminal pro-brain natriuretic peptide (NT-proBNP) is a well-recognized biomarker for cardiac dysfunction and mortality. The association of NT-proBNP with mortality in MHD has been shown by numerous studies [4,5]. Soluble suppression of tumorigenicity 2 (sST2) is a regulatory factor closely related to inflammation [6], and has been reported associated with mortality and fatal cardiovascular events in dialysis patients [7,8]. Moreover, circulating sST2 has been recommended as an additional risk stratification factor for acute and chronic heart failure by the American Heart Association [9]. Although some researches have investigated the predicters for adverse outcomes in MHD patients, there is a lack of studies evaluating the prediction ability of the same factors to both short-term and long-term outcomes. Previous researches revealed that the influence of comorbid conditions on mortality could change over time [5,10]. Though previous studies showed that both NT-proBNP and sST2 were able to predict poor outcomes of MHD patients, whether they are both predictive of short-term and long-term outcomes remains unclear. The current research aims to explore the predictive ability of serum sST2 and NT-proBNP for short-term and long-term mortality and to provide evidence for their detection value in MHD patients.

## Materials and methods

### Study population and design

213 patients underwent regular hemodialysis treatment in July 2014 at the Ruijin Blood Purification Center consented to participate in the study. 8 patients who were over 85 years old, whose dialysis vintage was less than 3 months, who had an unstable dry weight, who had a history of myocardial infarction or active infection within 3 months were excluded. In total, 205 patients underwent serum sST2 detection. The patients were followed up continuously until July 2017. The date of outcomes was recorded. Fatal events and other clinical events, including transferring to other dialysis centers or receiving kidney transplantation, were recorded. The primary outcome of the current study was all-cause death, and the secondary outcome was fatal cardiovascular events, which included acute myocardial infarction, acute congestive heart failure, lethal cardiac arrest, stroke, secondary epilepsy and unexpected death defined as patients being found dead in their sleep by family members. The flow chart of the study is presented in Supplementary Figure 1.

### Data collection and laboratory measurements

The patients’ medical history, demographic characteristics, medications and dialytic prescriptions were collected during the recruitment period. Predialysis biochemical indicators (e.g., hemoglobin (Hb), albumin (ALB), creatinine (SCr), blood urea nitrogen (BUN), serum iron (SI), serum ferritin (SF), C-reactive protein (CRP) and NT-proBNP) and postdialysis BUN, were measured in the central clinical laboratory of Ruijin Hospital and collected during the recruitment period.

The remaining predialysis blood serum samples were preserved at −80 °C for further detection. Patients’ serum sST2 levels were detected by Human ST2/IL-33R Quantikine ELISA Kit (R&D Systems, Minneapolis, MN). Each sample was tested twice in twin wells to calculate the mean value as the final level of serum sST2. The detection range of the serum sST2 immunoassay was 6.74–20.42 ng/ml. The intra-assay precision was 4.4–5.6%, and the interassay precision was 5.4–7.1%. No significant cross-reactivity or interference was observed. The formulas for body mass index (BMI), urea reduction ratio (URR), and K_t_/V are listed in the Supplementary Material.

### Color Doppler echocardiography examinations

Patients underwent the color Doppler echocardiography examinations to evaluate cardiac function. The examination was performed by color Doppler diasonograph (Siemens, Acuson Sequoia C512, Germany) with a probe frequency of 3.5 MHz, left ventricular ejection fraction (LVEF), left ventricular end diastolic diameter (LVEDD), interventricular septal thickness (IVST) and left ventricular posterior wall thickness (PWT) were collected. The formulas for left ventricular mass (LVM), body surface area (BSA) and left ventricular mass index (LVMI) are listed in the Supplementary Material.

### Statistical analysis

The normality test was performed by the Kolmogorov–Smirnov test. A Q–Q plot was assessed visually to validate the normal distribution of the residuals. Continuous data with a normal distribution are presented as the mean ± SD. The comparison between groups was performed with two independent sample *t*-tests or one-way ANOVA, and Pearson’s correlation was used to analyze the association between sST2 and other clinical parameters. Nonnormally distributed data are presented as the median (interquartile range). The comparison between groups was performed by a nonparametric test, and binary correlation analysis was performed by Spearman’s correlation. Enumeration data are presented as X%, and the *χ*^2^ test was adopted to make comparisons between groups.

Univariate and multivariate linear regression analyses were used to analyze the association of relevant clinical factors with serum sST2. Variables with *p* < 0.1 in the univariate analysis were included in the multivariate model. Multivariate linear regression analysis was performed in the stepwise mode. All data analyses were performed with SPSS version 23.0 (SPSS Inc. Chicago, IL) and *p* values <0.05 were significant.

### Survival analysis

Short-term outcomes were defined as death within 1 year after patients enrolled, while long-term outcomes were defined as death within 3 years. The risk factors of short-term outcomes and long-term outcomes were analyzed separately. The Kaplan–Meier analysis was conducted to determine the discrepancy in mortality among patients with different levels of serum sST2 or NT-proBNP. The Kaplan–Meier curves were created according to the tertiles of each biomarker. Patients who were transferred to other dialysis centers or received kidney transplantation were considered survivors when conducting survival analysis.

To analyze the predictive ability of the two biomarkers for short-term and long-term outcomes in MHD patients, univariate and multivariate Cox regression analysis were performed to calculate hazard ratios and corresponding 95% confidence intervals (CIs). Apart from serum sST2 and NT-proBNP, several acknowledged variables were included in the multivariate hazard models, including sex, age, dialysis vintage, spKt/V, ALB and BMI, LVEF and LVMI.

## Results

### Baseline clinical characteristics

Among 205 MHD patients in the cohort, 61.0% were male. The median age was 59 (50, 68) years, the median dialysis vintage was 54.2 (30.7, 88.9) months, the median dry weight was 60.00 (50.50, 68.00) kg, the median intradialytic weight gain was 2.80 (2.00, 3.45) kg, the mean BMI was 21.78 ± 3.45 kg/m^2^, and the median spKt/V was 1.45 (1.29, 1.64). Approximately 95.1% of patients dialyzed through arteriovenous fistula, and 88.8% of patients received dialysis three times a week. Chronic glomerulonephritis was the leading cause of ESRD (58.0%). More than 90% of patients had hypertension, and 29.3% of patients had diabetes mellitus. The baseline laboratory results are presented in Supplementary Table 1.

#### Serum sST2 level

**Figure 1.**
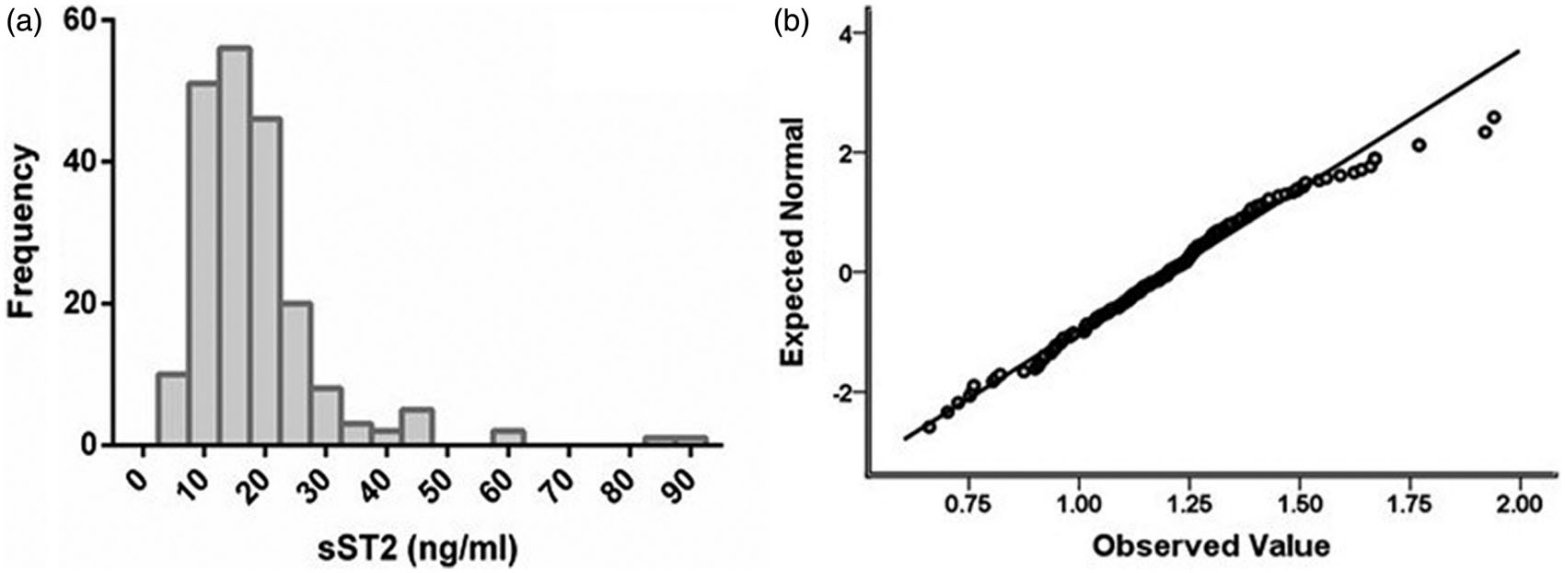
The histogram of serum sST2 level and normal Q–Q plot of LgsST2. (a) Histogram of sST2 level. (b) Normal Q–Q plot of LgsST2.

The median serum sST2 level was 15.99 (11.60, 20.49) ng/ml with non-normal distribution (Figure 1(a)), and Q–Q plot for LgsST2 was shown in Figure 1(b). The level of LgsST2 is 1.20 ± 0.21. No significant effect of drug utilization on serum sST2 was observed (Supplementary Table 2). Pearson correlation analysis showed that LgsST2 was significantly correlated to LgNT-proBNP (*r* = 0.257, *p* < 0.001), LgLVMI (*r* = 0.193, *p* = 0.006), BMI (r=-0.157, *p* = 0.024) and ALB (r=-0.147, *p* = 0.036).

### The association of serum sST2 and NT-proBNP with short-term outcomes in MHD patients

**Figure 2.**
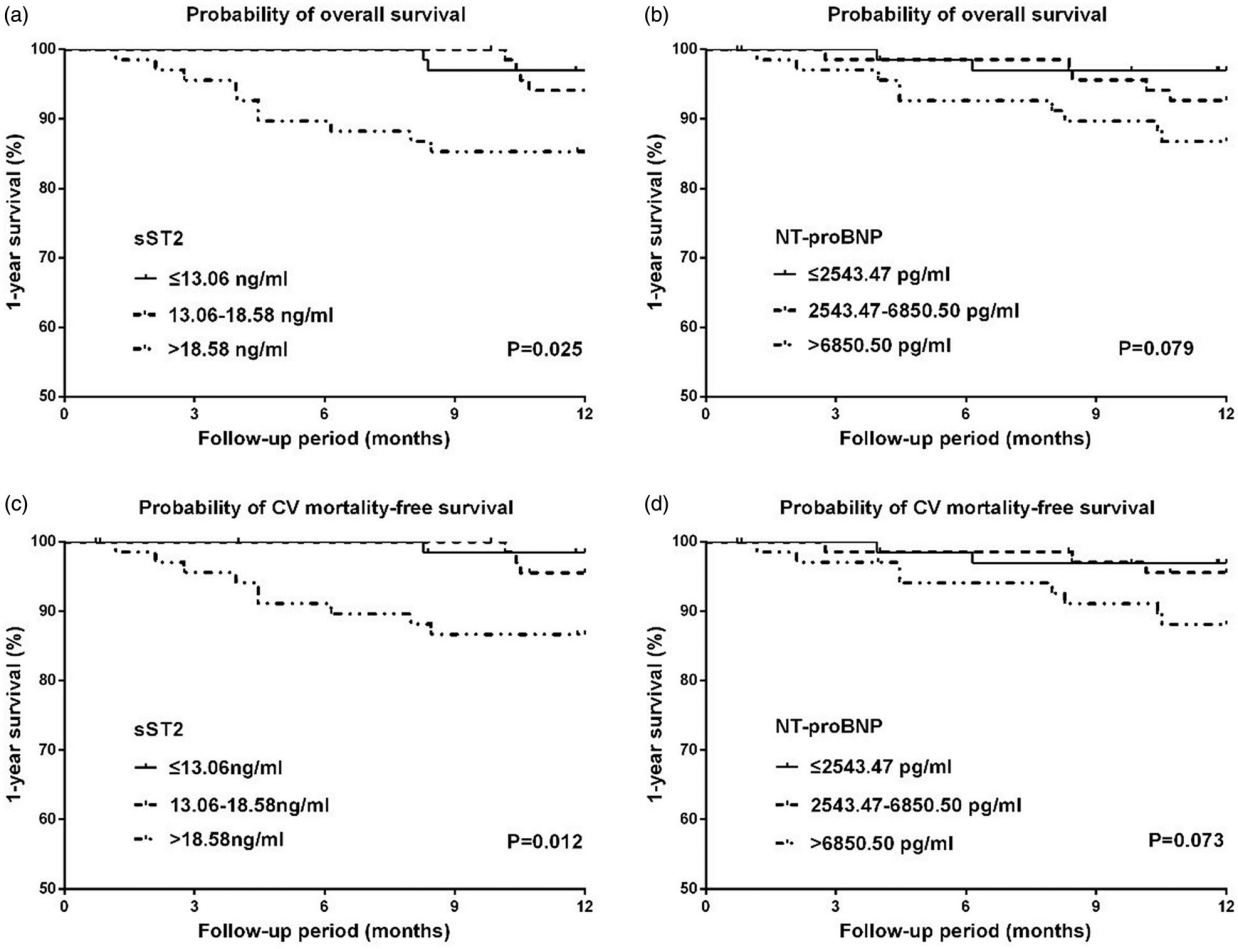
Kaplan–Meier curves for 1-year overall and cardiovascular mortality-free survival in patients stratified by tertiles of serum ST2 and NT-proBNP. (a) 1-year overall survival in patients stratified by tertiles of sST2, (b) 1-year overall survival in patients stratified by tertiles of NT-proBNP, (c) 1-year cardiovascular mortality-free survival in patients stratified by tertiles of sST2, (d) 1-year cardiovascular mortality-free survival in patients stratified by tertiles of NT-proBNP.

16 (7.8%) patients died during the 1-year follow-up, and 13 deaths (81.2%) were attributed to cardiovascular causes. The K–M analysis showed that both all-cause and cardiovascular mortality within 1 year rose significantly with increasing serum sST2 (*p* = 0.025 for mortality vs. *p* = 0.012 for CV mortality), while no significant difference was observed with increasing NT-proBNP (*p* = 0.079 for mortality vs. *p* = 0.073 for CV mortality) (Figure 2). In the Cox regression model, only sST2 was independently associated with both short-term all-cause mortality (adjusted HR = 1.036, *p* = 0.003) and cardiovascular mortality (adjusted HR 1.040, *p* = 0.001). No significant was shown in NT-proBNP (Table 1).

**Table 1.** Cox regression analysis of 1-year mortality.

Terms	Univariate		Model 1		Model 2	
	HR (95%CI)	*p*	HR (95%CI)	*p*	HR (95%CI)	*p*
All-cause mortality						
Male	1.592 (0.597, 4.2414)	0.353	–	NS	–	NS
Age	1.043 (1.000, 1.088)	0.049	–	NS	–	NS
Dialysis vintage	1.000 (0.991, 1.010)	0.97	–	NS	–	NS
SpKT/V	1.177 (0.207, 6.697)	0.854	–	NS	–	NS
BMI	0.831 (0.700, 0.987)	0.035	–	NS	–	NS
ALB	0.712 (0.634, 0.799)	<0.001	0.739 (0.656, 0.832)	<0.001	0.739 (0.656, 0.832)	<0.001
LVEF	0.956 (0.905, 1.029)	0.279	–	NS	–	NS
LVMI	1.007 (0.996, 1.019)	0.218	–	NS	–	NS
sST2	1.045 (1.024, 1.067)	<0.001	1.036 (1.012, 1.060)	0.003	1.036 (1.012, 1.060)	0.003
LgNT-proBNP	3.943 (1.443, 10.773)	0.007	/	/	–	NS
Cardiovascular mortality						
Male	1.360 (0.457, 4.048)	0.58	–	NS	–	NS
Age	1.040 (0.993, 1.089)	0.095	–	NS	–	NS
Dialysis vintage	1.002 (0.992, 1.012)	0.707	–	NS	–	NS
SpKT/V	0.779 (0.106, 5.726)	0.806	–	NS	–	NS
BMI	0.875 (0.729, 1.050)	0.151	–	NS	–	NS
ALB	0.735 (0.648, 0.834)	<0.001	0.767 (0.673, 0.875)	<0.001	0.767 (0.673, 0.875)	<0.001
LVEF	0.941 (0.882, 1.004)	0.066	–	NS	–	NS
LVMI	1.010 (0.998, 1.022)	0.119	–	NS	–	NS
sST2	1.048 (1.026, 1.071)	<0.001	1.040 (1.016, 1.065)	0.001	1.040 (1.016, 1.065)	0.001
LgNT-proBNP	4.487 (1.448, 13.910)	0.009	/	/	–	NS

When NT-proBNP was used in the Cox regression model, its HR and 95% CI for different dependent variant were all close to 1.000, therefore LgNT-proBNP was used in the Cox regression model for analysis.

Model 1: Adjusted by sex, age, dialysis vintage, spKT/V, BMI, ALB, LVEF, LVMI and sST2, Forward LR.

Model 2: Adjusted by sex, age, dialysis vintage, spKT/V, BMI, ALB, LVEF, LVMI, sST2 and LgNT-proBNP, Forward LR.

### The association of serum sST2 and NT-proBNP with long-term mortality in MHD patients

**Figure 3.**
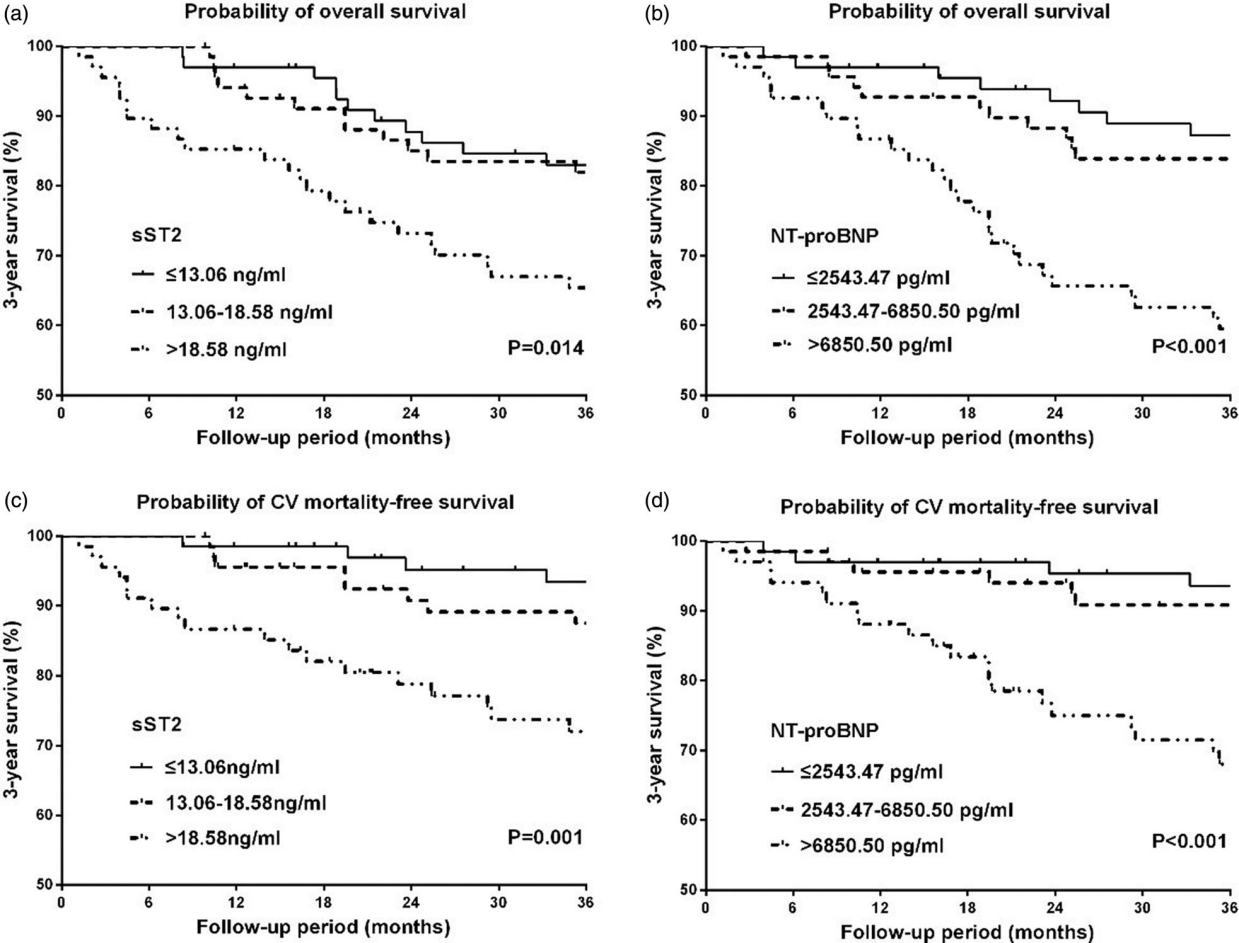
Kaplan–Meier curves for 3-year overall and cardiovascular mortality-free survival in patients stratified by tertiles of serum ST2 and NT-proBNP. (a) 3-year overall survival in patients stratified by tertiles of sST2, (b) 3-year overall survival in patients stratified by tertiles of NT-proBNP, (c) 3-year cardiovascular mortality-free survival in patients stratified by tertiles of sST2, (d) 3-year cardiovascular mortality-free survival in patients stratified by tertiles of NT-proBNP.

During the 3-year follow-up, 46 (22.4%) patients died in total, and 30 deaths (65.2%) were attributed to cardiovascular causes. The K-M analysis showed that both all-cause and cardiovascular mortality within 3 year rose significantly with increasing serum sST2 (*p* = 0.014 for mortality vs. *p* = 0.001 for CV mortality), as well as NT-proBNP (*p* < 0.001 for mortality vs. *p* < 0.001 for CV mortality) (Figure 3). In Cox regression analysis, sST2 (adjusted HR 1.020, *p* = 0.038) and LgNT-proBNP (adjusted HR 2.301, *p* = 0.022) were significant risk factors for long-term all-cause mortality, as well as cardiovascular mortality (sST2, adjusted HR 1.022, *p* = 0.034; LgNT-proBNP, adjusted HR 4.798, *p* < 0.001) (Table 2).

**Table 2.** Cox regression analysis of 3-year mortality.

Terms	Univariate		Model 2		Model 3	
	HR (95%CI)	*p*	HR (95%CI)	*p*	HR (95%CI)	*p*
All-cause mortality						
Male	1.004 (0.555, 1.815)	0.989	–	NS	–	NS
Age	1.042 (1.016, 1.068)	0.001	1.034 (1.010, 1.059)	0.005	1.034 (1.009, 1.060)	0.006
Dialysis vintage	1.001 (0.996, 1.007)	0.615	–	NS	–	NS
SpKT/V	0.341 (0.109, 1.066)	0.064	–	NS	–	NS
BMI	0.938 (0.857, 1.026)	0.163	–	NS	–	NS
ALB	0.818 (0.740, 0.904)	<0.001	0.828 (0.751, 0.913)	<0.001	0.837 (0.763, 0.918)	<0.001
LVEF	0.924 (0.892, 0.957)	<0.001	0.925 (0.893, 0.959)	<0.001	0.947 (0.910, 0.986)	0.008
LVMI	1.008 (1.001, 1.015)	0.027	–	NS	–	NS
sST2	1.033 (1.016, 1.049)	<0.001	1.028 (1.009, 1.047)	0.004	1.020 (1.001, 1.040)	0.038
LgNT-proBNP	3.765 (2.080, 6.814)	<0.001	/	/	2.301 (1.126, 4.701)	0.022
Cardiovascular mortality						
Male	1.904 (0.430, 1.899)	0.79	–	NS	–	NS
Age	1.053 (1.020, 1.087)	0.002	1.048 (1.016, 1.080)	0.003	1.045 (1.012, 1.079)	0.007
Dialysis vintage	1.004 (0.998, 1.010)	0.183	1.000 (1.006, 1.012)	0.047	–	NS
SpKT/V	0.437 (0.102, 1.873)	0.265	–	NS	–	NS
BMI	0.953 (0.854, 1.063)	0.389	–	NS	–	NS
ALB	0.802 (0.715, 0.900)	<0.001	0.823 (0.735, 0.920)	0.001	0.837 (0.755, 0.927)	0.001
LVEF	0.928 (0.888, 0.970)	0.001	0.927 (0.886, 0.970)	0.001	–	NS
LVMI	1.012 (1.004, 1.020)	0.003	–	NS	–	NS
sST2	1.038 (1.020, 1.056)	<0.001	1.035 (1.014, 1.057)	0.001	1.022 (1.002, 1.043)	0.034
LgNT-proBNP	5.212 (2.446, 11.106)	<0.001	/	/	4.798 (2.101, 10.957)	<0.001

Model 1: Adjusted by sex, age, dialysis vintage, spKT/V, BMI, ALB, LVEF, LVMI and sST2, Forward LR.

Model 2: Adjusted by sex, age, dialysis vintage, spKT/V, BMI, ALB, LVEF, LVMI, sST2 and LgNT-proBNP, Forward LR.

## Discussion

Our study is a single-center prospective cohort study. We included 205 patients under dialysis, and investigated the predicted value of serum sST2 and NT-proBNP for both shot-term and long-term outcomes. The main finding of our study was serum sST2 level was the only independent risk factor for short-term mortality. This finding suggested that the prognostic value of sST2 for short-term mortality may be better than that of NT-proBNP.

ST2 is a member of the interleukin-1 (IL-1) receptor family of glycoproteins produced by structural cells and epithelia cells. It is mainly expressed in two isoforms: the transmembrane form ST2L and the soluble form sST2. Extracellular sST2 plays an important role in promoting inflammation [11] and cardiac fibrosis [12] by blocking the protective effect of the IL-33/ST2L signaling pathway as a decoy receptor [13]. Bao et al. [12] found a significant increase in serum sST2 in patients with renal failure; however, the ascensional range of sST2 was significantly less than that of small molecular proteins, similar to that in other inflammatory chronic diseases, because the increment of serum sST2 in ESRD patients mainly stemmed from tractive myocardium and activated immune cells instead of descending from renal excretion.

Recent studies found that sST2 was engaged in the regulation of diverse cardiovascular diseases such as myocardial infarction [14], congestive heart failure [15,16] and cardiac hypertrophy [17] by activating inflammation. The latest guidelines of the American Heart Association recommended sST2 as an additional risk stratification factor for heart failure, especially in the acute phase [9]. MHD patients may be a target population for sST2 due to a high prevalence rate of cardiovascular morbidities, a higher level of sST2 [12,18] and persistent circumstances of microinflammation [19]. We also observed that both 1-year and 3-year all-cause and cardiovascular mortality rose significantly with serum sST2. Further analyses demonstrated that serum sST2 was an independent risk factor for both all-cause and cardiovascular mortality in the short-term and the long-term.

Due to the similarities of sST2 and NT-proBNP in identifying individuals with a higher risk of cardiovascular diseases and adverse clinical outcomes, comparisons have been made for these two biomarkers in recent years [20–22]. The pros and cons of sST2 and NT-proBNP are currently inconclusive. Two recent prospective clinical studies found that the effect of sST2 and NT-proBNP was different in predicting adverse outcomes in MHD patients. Multivariate Cox regression analysis reported by Obokata et al. revealed that only sST2 was an independent risk factor for 2-year mortality when sST2 and NT-proBNP were included in the regression model simultaneously [7]. Another study demonstrated that the prognostic power of sST2 to predict 3-year adverse clinical outcomes was weaker than that of NT-proBNP [8]. Apart from the discrepancy, neither of the two studies systematically investigated the prognostic value of the two biomarkers for short-term or long-term mortality in MHD patients; thus, the findings of our study may have an important impact on the clinical application of the two biomarkers. In this study, we found that 1-year mortality rose significantly with serum sST2 but not NT-proBNP, and sST2 was the only independent risk factor for 1-year mortality.

In addition, sST2 was a more stable biomarker in MHD patients due to the characteristic that it was less susceptible to dialysis treatment than NT-proBNP [23,24], which was cleared mainly by the kidney [25]. And serum sST2 has been considered as an acute phase protein in acute infections [6] and acute inflammatory diseases [26]; thus, it is closely associated with the level of recent inflammation activation. Considering the reasons above, circulating sST2 may be a better biomarker to reflect the near-term level of inflammation and myocardium traction in MHD patients. In contrast, though sST2 manifested a prognostic value for long-term mortality as well, its hazard ratio was relatively limited compared to that of NT-proBNP. Therefore, to enhance the prognostic value of sST2 for the long-term prognosis of our patients, the repetitive detection of serum sST2 may have high significance.

The strength of our study is to establish the prognostic application of sST2 for predicting the short-term mortality in MHD patients. MHD patient with higher sST2 level may have a higher mortality in one-year follow-up. Nephrologist could search for more detailed cardiovascular disease in these MHD patients, and more aggressive treatment and evaluation for cardiovascular disease may improve the prognosis of MHD patients. Our study also adds more evidence in the testing value of sST2 in MHD patients. Future studies could focus on whether lowering the sST2 level could improve the outcome.

## Limitations

There were a few limitations to the current study. First, this is a small-size single-center study, and we enroll the stable hemodialysis patients. We exclude initiated hemodialysis patients, so we could not analyze and compare the performance of sST2 and NT-proBNP in predicting 3-month death in new HD patients. However, we believe excluded new HD patients may decrease confounding factors for the study. Second, the number of patients who died in the first year was limited (16 patients died within one year); therefore, the analysis of the short-term outcomes was easily influenced by selection bias. Finally, some patients were transmitted to other blood purification centers or received kidney transplantation. These withdrawn patients may also slightly influence the results of the survival analysis.

## Conclusions

Serum sST2 is a novel biomarker associated with adverse clinical outcomes. We confirmed the significant prognostic value of serum sST2 for both all-cause and cardiovascular mortality in MHD patients. The findings of the current research suggest that the measurement of sST2 may provide better prognostic value in short-term prognosis than the classic biomarker NT-proBNP. Further large-scale, multicenter and prospective studies are needed in the future.

## Supplementary Material

Supplemental Material

Supplemental Material

## Data Availability

The datasets generated during and/or analyzed during the current study are available from the corresponding author on reasonable request.

## Abbreviations

sST2: Suppression of Tumorigenicity 2
NT-proBNP: N-terminal pro-brain natriuretic peptide
MHD: Maintenance hemodialysis
IL-33: Interleukin-33
Hb: Hemoglobin
ALB: Albumin
Scr: Serum creatinine
UA: Uric acid
Ca: Calcium
P: Phosphate
iPTH: intact parathyroid hormone
25(OH)D: 25-hydroxy vitamin D
SI: serum iron
SF: serum ferritin
CRP: C-reactive protein
NT-proBNP: N-terminal pro-brain natriuretic peptide
BMI: Body Mass Index
URR: Urea nitrogen reduction rate
spKt/V: Single-pool Kt/V
IL-1: Interleukin-1
