# Supplementary material for: Superior prognostic value of soluble suppression of tumorigenicity 2 for the short-term mortality of maintenance hemodialysis patients compared with NT-proBNP: a prospective cohort study
Source: Ren Fail. 2020 May 27;42(1):523–30. doi: 10.1080/0886022X.2020.1767648 (PMC7337010; doi:10.1080/0886022X.2020.1767648)

Supplementary Figure 1. Experimental design to explore the roles of sST2 and NT-proBNP in predicting the short- and long-term mortality of maintenance hemodialysis (MHD) patients.

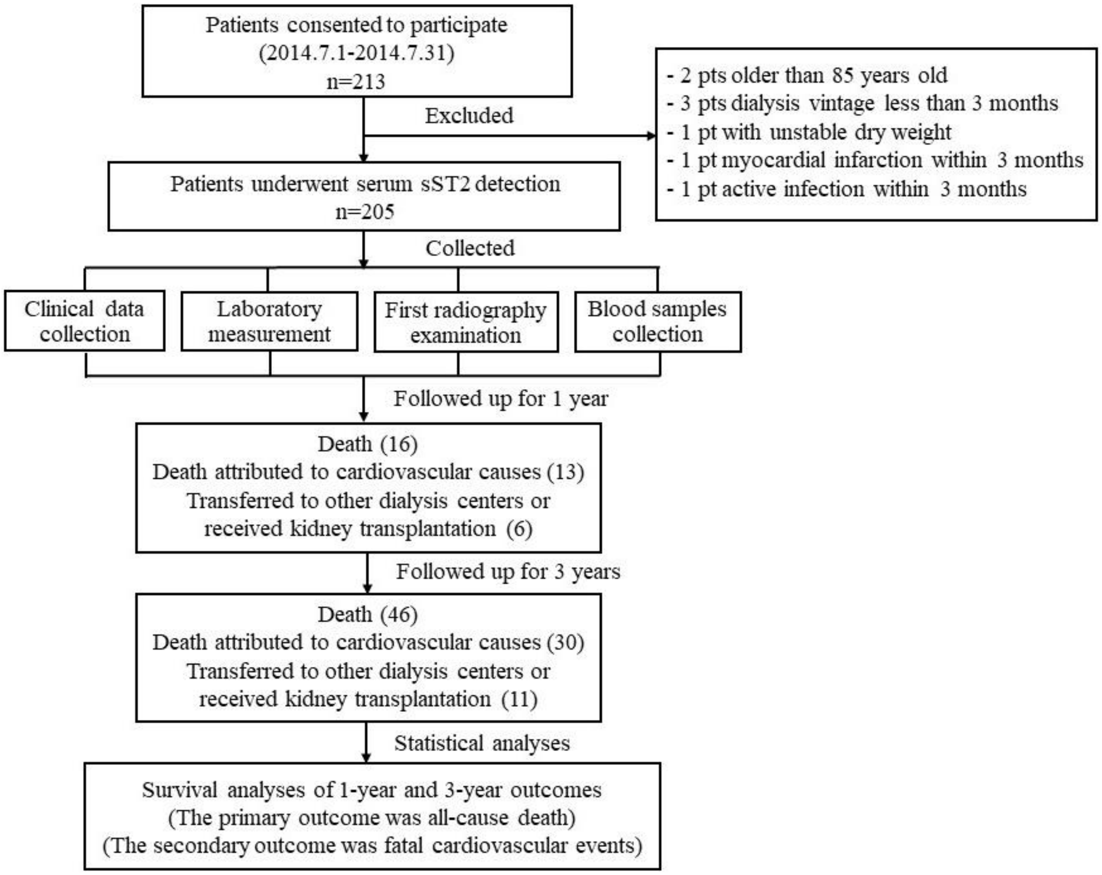

Supplement: Supplemental Material [file IRNF_A_1767648_SM4283.pdf]
