# Supplementary material for: Superior prognostic value of soluble suppression of tumorigenicity 2 for the short-term mortality of maintenance hemodialysis patients compared with NT-proBNP: a prospective cohort study
Source: Ren Fail. 2020 May 27;42(1):523–30. doi: 10.1080/0886022X.2020.1767648 (PMC7337010; doi:10.1080/0886022X.2020.1767648)
Supplement: Supplemental Material [file IRNF_A_1767648_SM4280.pdf]

**Supplementary**

***Calculation formula***

Body Mass Index (BMI)= Weight (kg)/Height$^2$(m$^2$).

Urea nitrogen reduction rate (URR)=(Post-dialysis BUN - Pre-dialysis BUN)/ Pre-dialysis BUN.

single-pool Kt/V (spKt/V) =$-\ln(\mathrm{R}-0.008\times\mathrm{T})+\frac{(4-3.5\times\mathrm{R})\times\mathrm{UF}}{W}$. R: Post-dialysis BUN/Pre-dialysis BUN, T: single dialysis duration (h), UF: ultrafiltration (L), W: dry weight (kg).

Left ventricular mass (LVM) (g)=0.8×1.04×[(LVEDD+IVST+PWT)$^3$-LVEDD$^3$]+0.6. LVEDD: Left ventricular end diastolic diameter (cm), IVST: Interventricular septal thickness (cm), PWT: Left ventricular posterior wall thickness (cm).

Body surface area (BSA) (m$^2$)=0.0061×Height (cm)+0.0128×Weight (Kg)-0.1529.

Left ventricular mass index (LVMI) (g/m$^2$)=LVM (g)/ BSA (m$^2$).

**Supplementary Table 1.** Baseline laboratory results of 205 recruited hemodialysis patients.

| Parameters | Total | Parameters | Total |
|---|---|---|---|
| Hb (g/dl) | 110.00 (103.00,117.00) | iPTH (pg/mL) | 261.60 (120.85,583.05) |
| ALB (g/L) | 34.00 (32.00,36.00) | 25(OH)D (nmol/L) | 54.75 (36.46,79.66) |
| Scr (μmol/L) | 976.66±244.44 | SI (μmol/L) | 9.95 (7.80,13.40) |
| UA (μmol/L) | 446.42±88.85 | SF (ng/mL) | 123.10 (53.80,262.30) |
| Ca (mmol/L) | 2.37±0.24 | CRP (mg/dl) | 0.35 (0.23,0.71) |
| P (mmol/L) | 1.90±0.57 | NT-proBNP (pg/mL) | 4062.00 (1962.00,9506.00) |
| LVEF (%) | 64 (60,68) | LVMI (g/m$^2$) | 116.16 (97.23, 146.67) |

Hemoglobin, Hb; Albumin, ALB; Serum creatinine, Scr; Uric acid, UA; Calcium, Ca; Phosphate, P; intact parathyroid hormone, iPTH; 25-hydroxy vitamin D, 25(OH)D; Serum iron, SI; Serum ferritin, SF; C-reactive protein, CRP; left ventricular ejection fraction, LVEF; left ventricular mass index, LVMI.

**Supplementary Table 2.** The influence of drug utility to LgsST2 concentration

| Drugs | LgsST2 (ng/ml) | T or F | P |
|---|---|---|---|
| CCB | | | |
| Yes (n=156) | 1.20±0.25 | -0.111 | 0.912 |

| | | | |
|---|---|---|---|
| No (n=49) | 1.20±0.20 | | |
| ACEI and/or ARB | | | |
| Both (n=39) | 1.19±0.23 | | |
| Either (n=102) | 1.18±0.21 | 2.065 | 0.129 |
| Neither (n=64) | 1.25±0.22 | | |
| β blocker | | | |
| Yes (n=88) | 1.19±0.20 | 0.794 | 0.428 |
| No (n=117) | 1.21±0.23 | | |
| α blocker | | | |
| Yes (n=71) | 1.16±0.20 | 1.975 | 0.05 |
| No (n=134) | 1.22±0.22 | | |
| Oral iron | | | |
| Yes (n=95) | 1.20±0.22 | 0.377 | 0.706 |
| No (n=110) | 1.21±0.21 | | |
| Intravenous iron | | | |
| Yes (n=25) | 1.20±0.25 | 0.16 | 0.873 |
| No (n=180) | 1.20±0.21 | | |
| ESA | | | |
| Yes (n=198) | 1.08±0.25 | -1.542 | 0.125 |
| No (n=7) | 1.21±0.21 | | |
| Calcium carbonate | | | |
| Yes (n=153) | 1.20±0.21 | 0.753 | 0.452 |
| No (n=52) | 1.22±0.24 | | |
| Active vitamin D | | | |
| Yes (n=134) | 1.20±0.20 | -0.087 | 0.931 |
| No (n=71) | 1.20±0.25 | | |

Calcium channel blocker: CCB, Angiotensin Converting Enzyme Inhibitors: ACEI, Angiotensin receptor blocker: ARB, Erythropoiesis stimulating agent: ESA.
